# High performance with fewer labels using semi-weakly supervised learning for pulmonary embolism diagnosis

**DOI:** 10.1038/s41746-025-01594-2

**Published:** 2025-05-07

**Authors:** Zixuan Hu, Hui Ming Lin, Shobhit Mathur, Robert Moreland, Christopher D. Witiw, Laura Jimenez-Juan, Matias F. Callejas, Djeven P. Deva, Ervin Sejdić, Errol Colak

**Affiliations:** 1 The Edward S. Rogers Department of Electrical and Computer Engineering, University of Toronto, Toronto, ON Canada; 2 Department of Medical Imaging, St Michael’s Hospital, Unity Health Toronto, 30 Bond St, Toronto, ON M5B 1W8 Canada; 3 Department of Medical Imaging, Faculty of Medicine, University of Toronto, Toronto, ON Canada; 4 Li Ka Shing Knowledge Institute, St Michael’s Hospital, Unity Health Toronto, Toronto, ON Canada; 5 Division of Neurosurgery, Department of Surgery, University of Toronto, Toronto, Canada; 6 North York General Hospital, Toronto, ON Canada

**Keywords:** Computed tomography, Cardiovascular diseases, Medical research, Computer science

## Abstract

This study proposes a semi-weakly supervised learning approach for pulmonary embolism (PE) detection on CT pulmonary angiography (CTPA) to alleviate the resource-intensive burden of exhaustive medical image annotation. Attention-based CNN-RNN models were trained on the RSNA pulmonary embolism CT dataset and externally validated on a pooled dataset (Aida and FUMPE). Three configurations included weak (examination-level labels only), strong (all examination and slice-level labels), and semi-weak (examination-level labels plus a limited subset of slice-level labels). The proportion of slice-level labels varying from 0 to 100%. Notably, semi-weakly supervised models using approximately one-quarter of the total slice-level labels achieved an AUC of 0.928, closely matching the strongly supervised model’s AUC of 0.932. External validation yielded AUCs of 0.999 for the semi-weak and 1.000 for the strong model. By reducing labeling requirements without sacrificing diagnostic accuracy, this method streamlines model development, accelerates the integration of models into clinical practice, and enhances patient care.

## Introduction

Machine learning (ML) shows great promise in transforming health care and medical imaging. Potential benefits include improved physician accuracy^1–4^, prioritization of examinations with critical findings^5–8^, helping mitigate radiologist shortages^9^, radiation dose reduction^10–12^, and improving image quality^13^. The training of medical imaging ML models has traditionally involved the annotation of large, curated datasets which is often a very resource-intensive exercise^14,15^. The annotation of such a large number of images can be a time-consuming and monotonous task, particularly for granular labels such as segmentation or bounding boxes. In addition, the recruitment of highly skilled expert radiologists as annotators can pose high financial costs as these professionals are in high demand, and their time is valuable. Ultimately, the time-consuming and resource-intensive nature of medical dataset annotation limits the scalability of a manual approach^16^. This is compounded by concerns over label accuracy, particularly in complex imaging studies. Employing multiple independent annotations aims to alleviate this, but challenges in interrater reliability persist^17^. Considering these challenges, there is growing interest in training models with less granular labels, semi-supervised techniques, and leveraging AI-assisted annotation. For example, less granular labels (e.g., exam rather than slice-level labels) can be extracted from radiologist reports via natural language processing or large language models^18^.

The Radiological Society of North America (RSNA) organizes annual AI challenges, necessitating significant effort in curating high-quality annotated medical imaging datasets. A prominent example is the RSNA cervical spine fracture CT dataset^19^, which provided three levels of labels: exam-level, cervical spine segment-level, and bounding box (pixel-level). Notably, the top-performing model from the cervical spine fracture detection challenge used only exam and segment-level labels, achieving remarkable performance without the detailed bounding box labels^20^. This observation suggests that models can excel even without utilizing highly granular labels. Additionally, researchers have explored the potential of weakly supervised learning techniques, such as multi-instance learning that use only exam-level labels. Studies on intracranial hemorrhage^21^ and COVID-19 detection on CT^22–24^ have demonstrated strong performance using solely exam-level labels, highlighting the potential of weakly supervised approaches in medical imaging.

The detection of pulmonary embolism (PE) on CT pulmonary angiography (CTPA) is a valuable use case for the investigation of label granularity and number in ML model development. PEs are blood clots in the pulmonary arterial circulation and a potentially life-threatening condition. PE can vary in dramatically in its presentation from large emboli occupying the central pulmonary arteries, to a small subsegmental embolus in the lung periphery. Larger PEs may span dozens of images, while smaller PEs may only occupy a small number of pixels within a few images. Detecting smaller PEs is challenging due to the large search space of the entire thorax covered by CTPA. Therefore, relying solely on exam-level labels may be insufficient, necessitating the use of more granular annotations.

Accurate and timely diagnosis of PE is essential to improving patient outcomes, as delays in diagnosis and intervention can significantly increase mortality. Without treatment, PE carries a mortality rate as high as 30%, compared to 8% with appropriate management^25^. Beyond immediate risks, PE complications can contribute to prolonged hospitalization and increased healthcare system costs^26^. Given its clinical significance and the diverse clinical and radiological presentations, PE represents a compelling use case for this study.

The wide variability in PE presentation allows us to take a closer look at the role of label granularity in model development. The RSNA Pulmonary Embolism CT Dataset (RSPECT)^27^ with slice and exam-level labels offers a valuable resource for exploring this. Recent research has primarily relied on detailed annotations like pixel or slice-level labels. For instance, Yang et al.^28^ and Shi et al.^29^ achieved notable results using pixel-level labels, while Huang et al.^30^ and Rajan et al.^31^ focused on slice-level annotations. Other studies, such as Suman et al.^32^ and Islam et al.^33^, explored full slice and exam-level labels. These studies collectively underscore the prevailing assumption that granular annotations are essential for accurate PE detection. This study challenges that assumption by investigating the potential of semi-weakly supervised learning.

The two most common types of supervised learning are strongly supervised and weakly supervised learning. In strongly supervised learning, models are trained on fully annotated data, such as using both slice-level and exam-level labels in the context of the RSPECT dataset. Weakly supervised learning, however, uses incomplete or less detailed annotations, often relying on coarser labels such as solely using exam-level labels. We introduce a third paradigm, semi-weakly supervised learning, which combines the broad coverage of exam-level labels with a strategically selected subset of slice-level annotations. Our hypothesis is that full slice-level annotations are not essential for good model performance. Instead, we suggest that a reduced number of slice-level labels can still yield comparable results to fully annotated models. By varying the proportion of slice-level annotations, we aim to identify a threshold that balances labeling efficiency with diagnostic accuracy. This approach can reduce the need for extensive hand-labeled data, potentially speeding up the development process for high-quality ML models in PE detection, and can be expanded to other medical imaging tasks. This could lead to significant cost savings and faster deployment of these models in clinical settings, ultimately benefiting patient care.

## Results

### Performance on overall PE detection

**Fig. 1:**
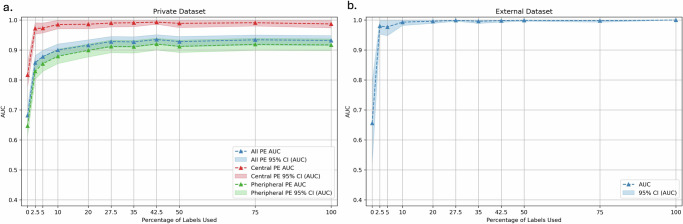
Impact of label granularity on model performance as a function of AUC. The graphs illustrate the performance of the models in terms of AUC across different datasets (**a**: RSPECT private test, **b**: external validation) as a function of the percentage of slice-level labels used. The solid lines represent the performance of average predictions across fivefold cross-validation, and the shaded areas correspond to the 95% confidence intervals (CI).

**Fig. 2:**
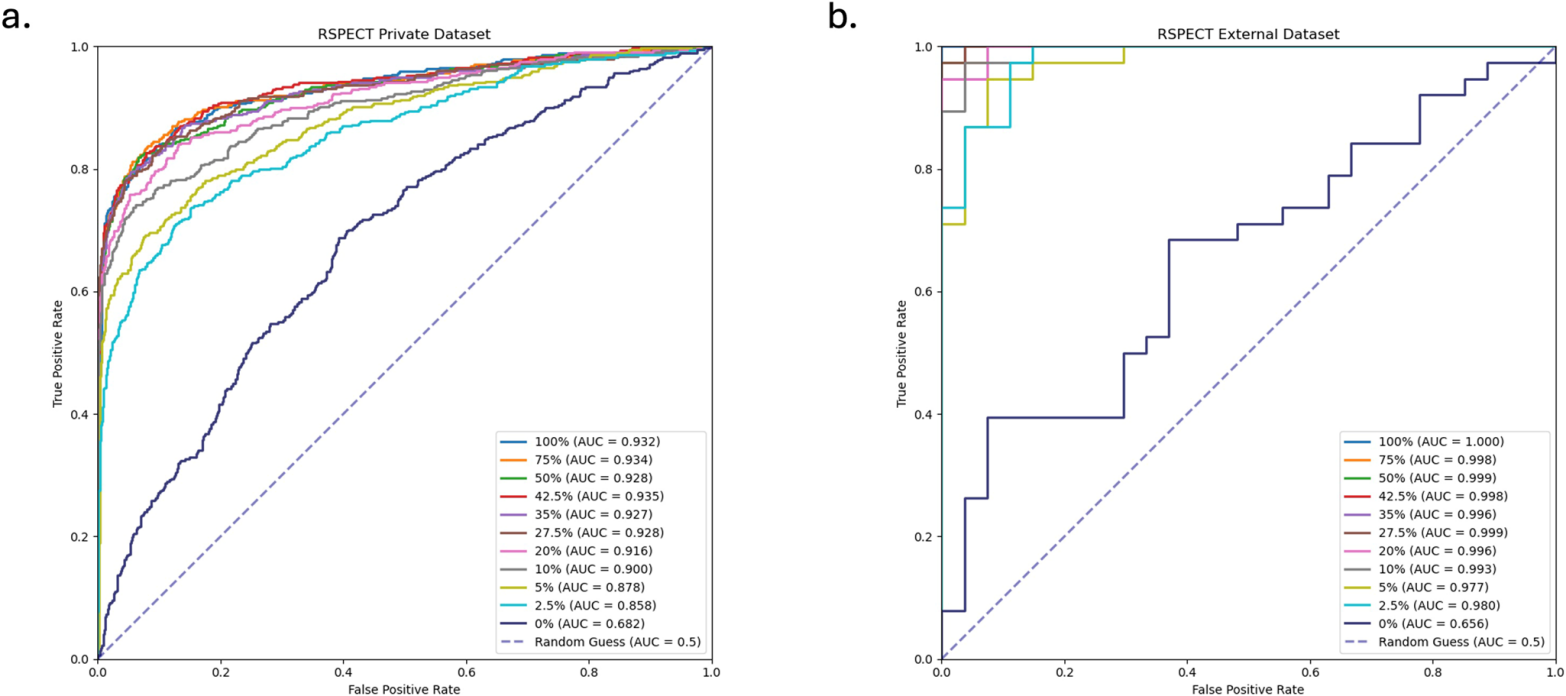
ROC Curves for all kinds of PE detection on the RSPECT private dataset. Receiver operating characteristic (ROC) curves for models trained with varying proportions of labeled data (0 to 100%) are shown for **a** the RSPECT private dataset and **b** a pooled external validation dataset. The corresponding area under the curve (AUC) values are displayed in the legend for each panel.

Model performance showed a significant initial improvement with just 2.5% of slice-level labels with the area under the receiver operating characteristic curve (AUC) increasing from 0.682 (0.652, 0.711) to 0.858 (0.836, 0.881) on the RSPECT private test set. Performance continued to improve with increasing label availability (Figs. 1, 2). However, beyond 27.5% label availability, the gains in performance became less substantial, showing marginal improvement (Table 1). For example, the AUC was 0.928 (0.910, 0.945) with 27.5% of slice-level labels compared to 0.932 (0.915, 0.948) when using all slice-level labels (*p* = 0.187).

**Table 1:** Performance in detecting PE on the RSPECT private test set

Slice Labels (%)	TP	FN	TN	FP	AUC	Acc	SEN	SEC	PPV	NPV	F1
0	351	87	437	569	0.682	0.546	0.801	0.434	0.382	0.834	0.517
2.5	313	125	871	135	0.858	0.820	0.715	0.866	0.699	0.874	0.707
5	305	133	912	94	0.878	0.843	0.696	0.907	0.764	0.873	0.729
10	344	94	860	146	0.900	0.834	0.785	0.855	0.702	0.901	0.741
20	327	111	955	51	0.916	0.888	0.747	0.949	0.865	0.896	0.801
27.5	344	94	954	52	0.928	0.899	0.785	0.948	0.869	0.910	0.825
35	351	87	942	64	0.927	0.895	0.801	0.936	0.846	0.915	0.823
42.5	364	74	915	91	0.935	0.886	0.831	0.910	0.800	0.925	0.815
50	356	82	942	64	0.928	0.899	0.813	0.936	0.848	0.920	0.830
75	367	71	921	85	0.934	0.892	0.838	0.916	0.812	0.928	0.825
100	350	88	939	67	0.932	0.893	0.799	0.933	0.839	0.914	0.819

*TP* true positive, *FN* false negative, *TN* true negative, *FP* false positive, *AUC* area under the receiver operating curve, *Acc* accuracy, *SEN* sensitivity, *SPEC* specificity, *PPV* positive predictive value, *NPV* negative predictive value.

Evaluations on the external dataset mirrored these findings (Table 2), showing similar improvements in AUC, accuracy, and F1 score with increasing label granularity. In particular, weakly supervised learning alone yielded a low AUC of 0.656 (0.522, 0.790), whereas adding only 2.5% of slice-level labels improved the AUC to 0.980 (0.953, 1.000), nearly matching the fully supervised model’s AUC of 1.000 (1.000, 1.000) (*p* = 0.124).

**Table 2:** Performance in detecting PE on the external validation set

Slice Labels (%)	TP	FN	TN	FP	AUC	Acc	SEN	SEC	PPV	NPV	F1
0	37	1	1	26	0.656	0.585	0.974	0.037	0.587	0.500	0.733
2.5	37	1	24	3	0.980	0.938	0.974	0.889	0.925	0.960	0.949
5	36	2	24	3	0.977	0.923	0.947	0.889	0.923	0.923	0.935
10	38	0	22	5	0.993	0.923	1.000	0.815	0.884	1.000	0.938
20	38	0	25	2	0.996	0.969	1.000	0.926	0.950	1.000	0.974
27.5	38	0	25	2	0.999	0.969	1.000	0.926	0.950	1.000	0.974
35	38	0	25	2	0.996	0.969	1.000	0.926	0.950	1.000	0.974
42.5	38	0	25	2	0.998	0.969	1.000	0.926	0.950	1.000	0.974
50	38	0	25	2	0.999	0.969	1.000	0.926	0.950	1.000	0.974
75	38	0	25	2	0.998	0.969	1.000	0.926	0.950	1.000	0.974
100	38	0	25	2	1.000	0.969	1.000	0.926	0.950	1.000	0.974

*TP* true positive, *FN* false negative, *TN* true negative, *FP* false positive, *AUC* area under the receiver operating curve, *Acc* accuracy, *SEN* sensitivity, *SPEC* specificity, *PPV* positive predictive value, *NPV* negative predictive value.

Detailed AUC values (95% CI) and *p* values from the DeLong test for various label percentages are provided in the Supplementary Materials (Supplementary Tables 1–4), as are results from the RSPECT public test set.

### Performance by PE subtype (central vs peripheral)

Figure 1 also shows the AUC curves for central and peripheral PE detection on the RSPECT private test set, with central PE results derived from the private central PE subset and peripheral PE results from the private peripheral PE subset. Similar to overall PE detection, both central and peripheral PE models benefited from increasing the proportion of slice-level labels. Detailed ROC curves are shown in Supplementary Fig. 1, and detailed comparisons are provided in Supplementary Table 5.

For central PE, the initial weakly supervised model (0% slice-level labels) already had a relatively high AUC of 0.817 (0.776, 0.858). Introducing just 2.5% of slice-level labels substantially improved the AUC to 0.972 (0.953, 0.991), closely approaching the fully supervised model’s AUC of 0.987 (0.974, 1.000) (*p* = 0.05).

In contrast, peripheral PE detection began with a lower baseline AUC of 0.647 (0.614, 0.680) under weakly supervised learning. Although adding 2.5% of slice-level labels improved performance to an AUC of 0.829 (0.802, 0.856), it required about 27.5% of slice-level labels to achieve near-peak performance (AUC 0.912 [0.891, 0.933]) close to the fully supervised model’s AUC of 0.917 (0.898, 0.937) (*p* = 0.119).

## Discussion

In this study, we investigated whether labeling every slice is necessary for accurate PE exam-level classification. Our experiments demonstrated that weakly supervised learning, using only exam-level labels, is limited for PE detection. The weakly supervised model achieved an AUC of just 0.682 (0.652, 0.711) on the RSPECT private test dataset, significantly lower than both strong and semi-weak learners. This is likely due to the need for localization of subtle emboli in PE diagnosis, which is more challenging than tasks like COVID-19 or intracranial hemorrhage (ICH) detection^21^ where weakly supervised methods have shown success^22–24^.

Detecting PE using CTPA can pose a significant challenge, even for experienced radiologists. Despite the high sensitivity of PE diagnosis on CTPA^34^, ML models should still possess the capability to detect smaller PE, such as subsegmental pulmonary embolism (SPE). SPEs are often small, occupying only a few voxels on imaging, akin to searching for a needle in a haystack. In fact, the positive predictive value of SPE diagnosis was a mere 25% when compared to the PIOPED II study^35^, underscoring the diagnostic complexity. Furthermore, interobserver agreement for SPE is notably lower compared to proximal PEs^36^. Additionally, filling artifacts may mimic true thrombotic material, adding to the complexity of the differentiation between PE and mimics^37^. Compounding these challenges are factors that contribute to poorer image quality, such as streak artifact, breathing motion, and poor opacification of the pulmonary arterial tree. These factors collectively degrade the sensitivity of PE detection, rendering it a considerably more challenging task compared to other pathologies that may require less granular annotation schema.

By incorporating a small number of labels, approximately 2.5% of total slice-level labels, we observed a significant performance boost. The AUC on the RSPECT private test dataset increased from 0.682 (0.652, 0.711) to 0.858 (0.836, 0.881), with similar improvements seen in the external validation dataset. Performance continued to improve with more slice-level labels but plateaued beyond 27.5% label availability, suggesting diminishing returns for additional labeling efforts.

**Fig. 3:**
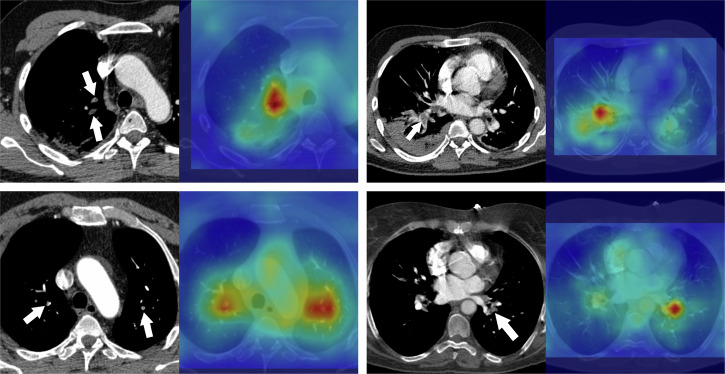
Example cases of model detected pulmonary embolism. Example cases highlighting a semi-weak learner accurately detecting pulmonary emboli in both peripheral and central locations. The left image component indicates the location of the PE (white arrows) while the right image component displays the model’s attention map using element wise Grad-CAM.

Our findings challenge the prevailing assumption in PE research that extensive fine-grained labeling is essential for high performance. Previous studies, such as those by Yang et al.^28^ and Shi et al.^29^, relied on detailed annotations and reported a sensitivity of 75.4% and an AUC of 0.812, respectively. Other approaches, like PENet by Huang et al.^30^, achieved an area under the receiver operator curves (AUROC) of 0.84 and 0.85 using slice-level labels, while Pi-PE by Rajan et al.^31^ reached an AUC of 0.85 on a dataset with predominantly segmental PE cases with sparsely annotated images. Islam et al.^33^ reported an AUC of 0.929 for exam-level PE using an ensemble model on 1000 cases from the RSPECT training dataset. In contrast, our semi-weakly supervised learner using only 27.5% of slice-level labels outperformed these prior studies, suggesting that a small but accurately labeled dataset can be sufficient, reducing the need for extensive labeling efforts. More detailed comparison, including label type, and the amount of each type of labels are presented in Table 3. Figure 3 showcases example images where the semi-weak learner demonstrated its ability to correctly detect small PE. Moreover, unlike these prior studies that often used smaller testing datasets, we evaluated our models on the RSPECT public test, RSPECT private test, and external test datasets with a total of over 2100 exams, providing a more robust examination of model performance.

**Table 3:** Summary of prior pulmonary embolism (PE) detection studies, highlighting differences in model architectures, data sources, annotation granularity, and labeling strategies, along with their reported performance metrics

Studies	Model type	Data used	Label types	Quantity of labels	Performance Metrics
Ref. ^28^	Two-stage 3D CNN + 2D CNN for false positive removal	PE challenge dataset (20 scans), in-house dataset (129 scans)	Pixel-level, region-level	Region-level annotations for 129 CTPA scans (own dataset); candidate voxel-level labels.	Sensitivity of 75.4% at 2 FPs/scan on PE Challenge dataset
Ref. ^29^	ResNet with attention loss + recurrent network	1670 sparsely annotated studies, 10,000 exam-level labels	Pixel-level, Exam-level	1670 pixel-level annotated studies; 10,000 exam-level labeled studies.	AUC 0.812 on a test set with 2160 studies
Ref. ^30^	3D CNN (end-to-end)	Internal (Stanford) dataset and external dataset (Intermountain)	Pixel-level	77-layer 3D CNN trained on volumetric annotations; results tested on two datasets: 169 internal and 200 external studies.	AUC 0.85 on external dataset
Ref. ^31^	Two-stage 2D Conv-LSTM for sparse annotated detection	Multi-hospital dataset, sparsely annotated at 10 mm spacing	Sparse pixel-level	Sparse contours at 10 mm slice spacing for 1874 positive and 718 negative studies.	AUC 0.94 on validation, 0.85 on high-severity test dataset
Ref. ^32^	CNN + Bi-LSTM with Attention Mechanism	RSPECT dataset (7279 studies), external test (106 studies)	Slice-level, Exam-level	Exam-level labels for 7279 studies (RSPECT dataset), tested on 106 external studies.	AUC 0.95 on external test dataset
Ref. ^33^	ViT, Swin transformer, CNNs with transfer learning and vessel-oriented image representation	RSPECT dataset (7279 studies), CAD-PE challenge, FUMPE, and in-house PE-CAD datasets	Slice-level, Exam-level	1,542,144 slices (train); Exam-level labels for 7279 studies.	AUC 0.929 on a hold-out test set from RSPECT training dataset with 1000 studies
Ours	CoAtNet + Attention + Bi-LSTM	RSPECT dataset (7279 studies), RSPECT public test dataset (650 studies), RSPECT private test dataset (1517 studies), CAD-PE Challenge, FUMPE	Slice-level, Exam-level	Partial slice labels depend on training strategy; Exam-level labels for all studies.	Semi-weak model: AUC 0.928 on private test set and AUC 0.999 for the pooled external validation dataset

In contrast to previous fully supervised or sparsely annotated approaches, our semi-weakly supervised model demonstrates competitive or superior performance while relying on fewer granular labels.

Our analysis of central versus peripheral PE detection further illustrates the variable need for granular labeling. Central PE, typically featuring larger and more conspicuous clots, proved easier to detect: with no slice-level labels, the model achieved an AUC of 0.817, and adding just 2.5% slice-level labels raised the AUC to 0.972, nearing the fully supervised model’s AUC of 0.987. In contrast, peripheral PE, often smaller and more subtle, started from a lower weakly supervised baseline AUC of 0.647 and required 27.5% of slice-level labels to reach near-peak performance (AUC 0.912 vs. fully supervised AUC 0.917). These findings suggest that while minimal granular labeling may suffice for “easier” tasks, more challenging cases—such as small or subsegmental PEs—benefit from additional granular annotations. A tiered or adaptive labeling strategy could thus be employed, allocating more detailed labels only to complex cases, thereby optimizing both annotation efficiency and model performance.

However, our approach has limitations. The threshold tuning method based on Youden’s J index does not take into account the clinical consequences of false negative and positive predictions nor the clot burden of false negative cases. Balancing sensitivity and specificity in a clinical context might require different weighting to minimize missed PE cases. Another limitation is the relatively small size of the external validation dataset, which may affect the generalizability of our findings. Additionally, CT studies were standardized to 184 slices based on average lung size to enhance GPU efficiency and lung coverage. This uniform approach might impact model learning due to down-sampling or over-sampling of CT images. Future research could explore varying slice lengths to optimize diagnostic accuracy.

In conclusion, our semi-weakly supervised model achieved performance comparable to a fully supervised approach while requiring granular labels for only about 50 slices per exam, approximately one-quarter of the total. This finding suggests that not all imaging tasks demand exhaustive annotation, and that strategically allocating a limited proportion of slice-level labels can still guide models toward robust diagnostic performance. By reducing the substantial labor and cost of manual labeling without compromising accuracy, our approach offers a resource-efficient, scalable path to integrating AI into clinical imaging workflows. Moreover, the observed differences in labeling requirements between central and peripheral PEs imply that future strategies may tailor annotation granularity based on lesion complexity. Overall, these insights promote more cost-effective and clinically impactful implementations of AI in medical imaging.

## Methods

### Dataset description

Ethics review board approval was not required as this study utilized publicly available, open-source data. Our study utilized the RSPECT dataset, which originally comprised 9,446 CTPA exams (Table 4) and was sourced from the Kaggle pulmonary embolism detection competition (https://www.kaggle.com/competitions/rsna-str-pulmonary-embolism-detection). In this competition, the data were partitioned into three non-overlapping subsets: training, public test, and private test. Following the competition’s protocol, we developed our models using the training set, performed model tuning on the public test set, and conducted final evaluations on the private test set. Additionally, we employed two publicly available datasets, Aida and FUMPE, which together provided 65 exams (38 positives) for external validation (Table 4)^38–40^.

**Table 4:** Demographics and label distribution for RSNA 2020 PE detection challenge (RSPECT) and external validation (Aida and FUMPE) datasets

Dataset	Split	Demographics			Slice-level labels			Examination level labels		
		Male	Female	Age *(y)*	Positive	Negative	Total	Positive	Negative	Total
**RSPECT**	*Train*	-	-	-	96,540 (5.39)	1,694,054 (94.61)	1,790,594	2368 (32.53)	4911 (67.47)	7279
	*Public*	-	-	-	7451 (5.07)	139,402 (94.93)	146,853	200 (30.76)	450 (69.24)	650
	*Private*	-	-	-	18,846 (4.89)	366,392 (95.11)	385,238	468 (30.85)	1049 (69.15)	1517
**External**	*Aida*	-	-	45–93	624 (6.16)	9498 (9.38)	10,122	5 (16.67)	25 (83.33)	30
	*FUMPE*	17	18	24–82	2304 (26.2)	6488 (73.8)	8792	33 (94.29)	2 (5.71)	35
	*Pooled*	NA	NA	24–93	2928 (15.5)	15,986 (84.5)	18,914	38 (58.46)	27 (41.54)	65

Data we presented as the number of labels with percentages in parentheses. Age is provided as a range.

**Fig. 4:**
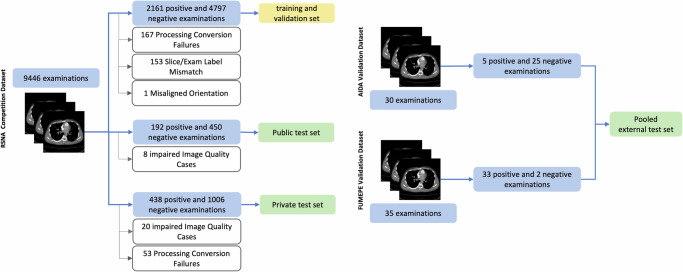
Data cleaning and splitting pipeline. This flowchart illustrates the preprocessing and splitting of the RSPECT, AIDA, and FUMPE datasets into various sets for internal training, validation, and testing. The white boxes highlight the exclusion criteria (label mismatches, processing failures, and misaligned orientations), the yellow box highlights the training dataset, and the green box highlights the test datasets.

For preprocessing, we performed extensive data cleaning. Digital imaging and communications in medicine (DICOM) files from the RSPECT dataset were converted to neuroimaging informatics technology initiative (NIfTI) format using the dicom2nifti Python library. We utilized TotalSegmentator^41^ to segment the lungs, thereby defining our three-dimensional volume of interest (VOI). Specifically, a 3D bounding box was generated to encompass the segmented lung region. Within this VOI, we applied window settings (width = 700, center = 100) to achieve optimal contrast and normalization of image intensities^42^. To ensure label consistency, we enforced the rule that an exam is labeled positive only if at least one slice within the exam is positive. During this validation, we identified and removed 153 examinations that were labeled as positive at the exam-level but contained no positive slice labels, indicating incoherent labeling. Additionally, we excluded exams labeled as indeterminate due to impaired image quality^27^. Detailed preprocessing steps are shown in Fig. 4, with additional exclusion reasons provided in Supplementary Table 6. This process distilled the RSPECT dataset to 6958 training examinations (2161 positives: 396 central PE and 1765 peripheral PE), 642 public test set examinations (192 positives: 39 centrals and 153 peripherals), and 1444 private test set examinations (438 positives: 89 centrals and 349 peripherals). The pooled external validation dataset was unchanged. The RSPECT public and private test sets and pooled external validation datasets were used for model evaluation.

The private test set was further divided into two subsets: the Central PE (main pulmonary arteries) dataset and the Peripheral PE (segmental or subsegmental pulmonary arteries) dataset, based on granular labeling. Both subsets included all 1,006 negative cases. The Central PE dataset comprised 89 positive cases labeled as “Central PE = = 1”, while the Peripheral PE dataset included 349 positive cases without the “Central PE = = 1” label. These subsets were designed to evaluate model performance in distinguishing between central and peripheral PE, further validating relationships between granular labels and different PE types.

### Data augmentation

Data augmentation was utilized to prevent overfitting. Using the Albumentations Python library^43^, we applied a suite of augmentations to enhance dataset diversity, including random rotation (0 to 10 degrees), scaling and translation (up to 10%), and modifications to image brightness and contrast. We also incorporated random horizontal flips, motion blur, median blur, Gaussian blur, and Gaussian noise (variance of 0.004). Additionally, we performed random cutouts and applied optical or grid distortions. Finally, we combined adjacent axial slices into a single 3-channel image for our models.

### Model architectures

**Fig. 5:**
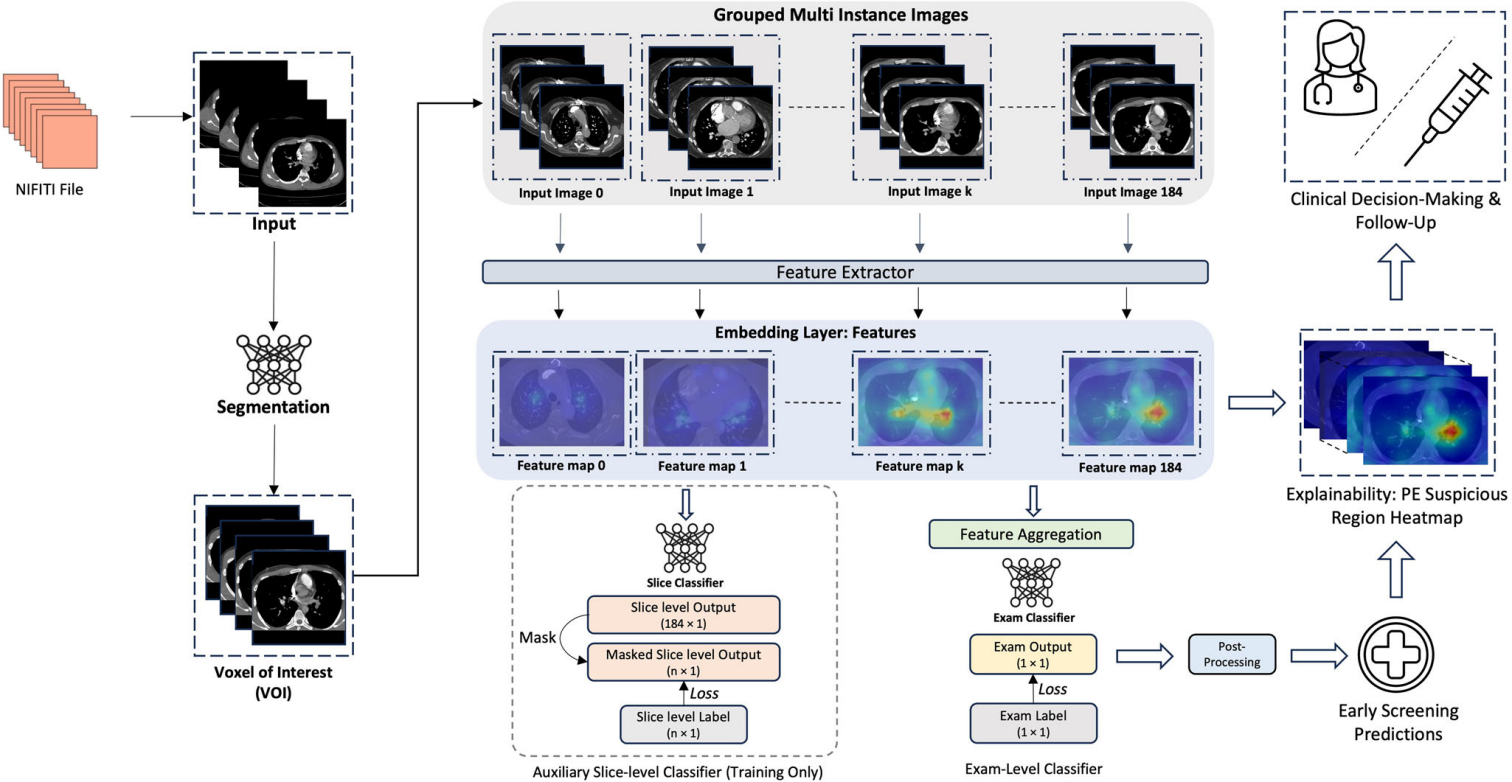
End-to-end training pipeline. This diagram illustrates the training pipeline for PE diagnosis using CoAtNet-0 as the feature extractor. The pipeline supports label granularity impact analysis by allowing slice-level classifier predictions to be masked. Strongly supervised learning (strong learner) uses all slice-level classifiers, while weakly supervised learning (weak learner) uses none. This setup enables experiments to assess the impact of varying amounts (*n*) of slice-level labels (0, 2.5, 5, 10, 20, 27.5, 35, 42.5, 50, 75, and 100%) on model performance.

We used an end-to-end training pipeline (Fig. 5) to develop three learners: weakly, strongly, and semi-weakly supervised (Supplementary Fig. 2). To implement these models, we utilized transfer learning with a CoAtNet-0 model^44^, pretrained on ImageNet and provided by HuggingFace^45^. The CoAtNet model, which combines convolutional networks and transformers, served as the feature extractor. We then applied batch normalization, an attention layer, and three bidirectional LSTM layers to aggregate features sequentially along the z-axis within CT scans. Finally, a fully connected layer outputted a probability score (0 to 1) after sigmoid normalization. Additional experiment results with alternative feature extractors (ViT and other CNNs) are included in the Supplementary Materials (Supplementary Fig. 3 and Supplementary Table 7).

To control the level of supervision, we introduced a hyperparameter that defined the proportion of slices retaining their instance-level labels, while the remainder were masked. To reflect real-world annotation practices, we chose to evenly sample the labeled axial slices from the extent of the lungs. This method ensures that annotations are uniformly distributed across the lung volume, mirroring how radiologists typically annotate images to capture diverse regions and variations. For example, if there were 200 lung slices and the proportion was 27.5%, ~54 slices retained their labels, and 146 were masked. At higher proportions, the model receives more fine-grained, slice-level guidance, making it more similar to a strongly supervised model. Conversely, at lower proportions, the model must rely more heavily on exam-level labels, closely simulating weaker supervision. Using all slices corresponded to a strongly supervised model, and using no slices corresponded to a weakly supervised model.

### Model training setup

We used the filtered training set of the RSPECT dataset, consisting of CT images resized to (184, 256, 256). To address the class imbalance, we implemented a weighted random sampling strategy, assigning weights of ~3.22 (total number of exams/number of positive examples = 6958/2161) to positive PE cases and 1.45 (total number of exams/number of negative examples = 6958/4797) to negative PE cases. These weights were applied using PyTorch’s WeightedRandomSampler in the DataLoader, ensuring each training epoch included a balanced representation of both classes.

All models were developed with PyTorch version 2.1.0 and trained on two NVIDIA A100 GPUs, each with 80 GB of memory. Training employed the Adam optimizer with an initial learning rate of 1e-4 and a batch size of 16. We used the binary cross-entropy loss function to evaluate performance. Each training run consisted of 30 epochs, incorporating early stopping to prevent overfitting and a Cosine Annealing learning rate scheduler (*T*_max_ = 30, min_lr = 1e-6) to dynamically adjust the learning rate.

### Experiment setup, evaluations, and statistical analysis

To analyze the impact of label granularity, we tested different percentages of slice-level labels used for model training. The weakly supervised model used only exam-level labels. The strongly supervised model used all exam and slice-level labels. Semi-weakly supervised models were trained with all exam-level labels and 0, 2.5, 5, 10, 20, 27.5, 35, 42.5, 50, 75, and 100% of available slice-level labels. To provide clearer context, these percentages roughly translate into the number of slice-level annotations needed per exam. For instance, utilizing 2.5% of slice-level labels corresponds to about 4 slice annotations per exam, whereas using 27.5% translates to ~53 slice annotations per exam (see Supplementary Table 8). Fivefold cross-validation (CV) was used for training. All evaluations were performed at the exam-level. Initial performance assessments were conducted using the hold-out RSPECT public test dataset. We used Youden’s J index to identify the optimal threshold on the receiver operator curve (ROC) by maximizing the difference between true and false positive rates. This threshold was applied to the evaluation using the RSPECT private test set. To assess generalizability, we performed external validation on pooled Aida and FUMPE datasets.

Model performances were primarily assessed based on average predictions across the five CV models using AUC, accuracy, sensitivity, specificity, positive predictive values (PPV), and negative predictive values (NPV). We further compared model performance by conducting pairwise AUC comparisons using the DeLong test. Confidence intervals were calculated with the confidence interval Python library (v1.0.4), employing the binomial method for accuracy, sensitivity, specificity, PPV, and NPV, and the fast DeLong method for AUC^46^.

## Supplementary information

Supplementary Materials

## Data Availability

The RSPECT training dataset is available via the competition page at https://www.kaggle.com/c/rsna-str-pulmonary-embolism-detection. External validation datasets are available at https://figshare.com/collections/FUMPE/4107803/1 and https://datahub.aida.scilifelab.se/10.23698/aida/ctpa.

## Code availability

The source code used in this study is available at https://github.com/zxjasonhu/pe_granular_analysis.

## References

[1] Bennani, S., et al. Using AI to improve radiologist performance in detection of abnormalities on chest radiographs. *Radiology* **309**, e230860 (2023). 38085079 10.1148/radiol.230860

[2] Lee, J. H. et al. Improving the performance of radiologists using artificial intelligence-based detection support software for mammography: a multi-reader study. *Korean J. Radiol.* **23**, 505–516 (2022). 35434976 10.3348/kjr.2021.0476 PMC9081685

[3] Eng, D. K. et al. Artificial intelligence algorithm improves radiologist performance in skeletal age assessment: a prospective multicenter randomized controlled trial. *Radiology* **301**, 692–699 (2021). 34581608 10.1148/radiol.2021204021

[4] Tam, M. D. B. S. et al. Augmenting lung cancer diagnosis on chest radiographs: positioning artificial intelligence to improve radiologist performance. *Clin. Radiol.* **76**, 607–614 (2021). 33993997 10.1016/j.crad.2021.03.021

[5] Topff, L., et al. Artificial intelligence tool for detection and worklist prioritization reduces time to diagnosis of incidental pulmonary embolism at CT. *Radiol. Cardiothorac. Imaging* **5**, e220163 (2023). 37124638 10.1148/ryct.220163 PMC10141443

[6] Prevedello, L. M. et al. Automated critical test findings identification and online notification system using artificial intelligence in imaging. *Radiology* **285**, 923–931 (2017). 28678669 10.1148/radiol.2017162664

[7] Annarumma, M. et al. Automated triaging of adult chest radiographs with deep artificial neural networks. *Radiology* **291**, 196–202 (2019). 30667333 10.1148/radiol.2018180921 PMC6438359

[8] Winkel, D. J., Heye, T., Weikert, T. J., Boll, D. T. & Stieltjes, B. Evaluation of an AI-based detection software for acute findings in abdominal computed tomography scans: toward an automated work list prioritization of routine CT examinations. *Invest. Radiol.* **54**, 55–59 (2019). 30199417 10.1097/RLI.0000000000000509

[9] Meng, F., Zhan, L., Liu, S. & Zhang, H. The growing problem of radiologist shortage: China’s perspective. *Korean J. Radiol.* **24**, 1046–1048 (2023). 37899513 10.3348/kjr.2023.0839 PMC10613839

[10] Ng, C. K. C. Artificial intelligence for radiation dose optimization in pediatric radiology: a systematic review. *Children* **9**, 1044 (2022). 35884028 10.3390/children9071044 PMC9320231

[11] Wang, Y. -R. J. et al. AI transformers for radiation dose reduction in serial whole-body PET scans. *Radiol. Artif. Intell.* **5**, e220246 (2023). 37293349 10.1148/ryai.220246 PMC10245181

[12] Jo, G. D. et al. 75% radiation dose reduction using deep learning reconstruction on low-dose chest CT. *BMC Med. Imaging* **23**, 121 (2023). 37697262 10.1186/s12880-023-01081-8 PMC10494344

[13] Tamada, D. Review: noise and artifact reduction for MRI using deep learning. Preprint at 10.48550/ARXIV.2002.12889 (2020).

[14] Willemink, M. J. et al. Preparing medical imaging data for machine learning. *Radiology* **295**, 4–15 (2020). 32068507 10.1148/radiol.2020192224 PMC7104701

[15] Kitamura, F. C. et al. Lessons learned in building expertly annotated multi-institution datasets and hosting the RSNA AI challenges. *Radiol. Artif. Intell.* **6**, e230227 (2024). 38477659 10.1148/ryai.230227 PMC11140499

[16] Tajbakhsh, N. et al. Embracing imperfect datasets: a review of deep learning solutions for medical image segmentation. *Med. Image Anal.* **63**, 101693 (2020). 32289663 10.1016/j.media.2020.101693

[17] Adams-McGavin, R. C. et al. Interrater agreement of CT grading of blunt splenic injuries: does the AAST grading need to be reimagined?. *Can. Assoc. Radiol. J.* **75**, 171–177 (2024). 37405424 10.1177/08465371231184425

[18] Wood, D. A. et al. Deep learning to automate the labelling of head MRI datasets for computer vision applications. *Eur. Radiol.* **32**, 725–736 (2022). 34286375 10.1007/s00330-021-08132-0 PMC8660736

[19] Lin, H. M. et al. The RSNA cervical spine fracture CT dataset. *Radiol. Artif. Intell.* **5**, e230034 (2023). 37795143 10.1148/ryai.230034 PMC10546361

[20] Flanders, A. et al. RSNA 2022 cervical spine fracture detection. Kaggle. https://kaggle.com/competitions/rsna-2022-cervical-spine-fracture-detection (2020).

[21] Teneggi, J., Yi, P. H. & Sulam, J. Examination-level supervision for deep learning–based intracranial hemorrhage detection on head CT scans. *Radiol. Artif. Intell.* **6**, e230159 (2024). 38294324 10.1148/ryai.230159 PMC10831525

[22] Chikontwe, P. et al. Dual attention multiple instance learning with unsupervised complementary loss for COVID-19 screening. *Med. Image Anal.* **72**, 102105 (2021). 34102477 10.1016/j.media.2021.102105 PMC8141701

[23] Han, Z. et al. Accurate screening of COVID-19 using attention-based deep 3D multiple instance learning. *IEEE Trans. Med. Imaging* **39**, 2584–2594 (2020). 32730211 10.1109/TMI.2020.2996256

[24] Wang, X. et al. A weakly-supervised framework for COVID-19 classification and lesion localization from chest CT. *IEEE Trans. Med. Imaging* **39**, 2615–2625 (2020). 33156775 10.1109/TMI.2020.2995965

[25] Bĕlohlávek, J., Dytrych, V. & Linhart, A. Pulmonary embolism, part I: epidemiology, risk factors and risk stratification, pathophysiology, clinical presentation, diagnosis and nonthrombotic pulmonary embolism. *Exp. Clin. Cardiol.* **18**, 129–138 (2013). 23940438 PMC3718593

[26] Imura, M., Yamamoto, T. & Hiasa, K. -I. Pulmonary thromboembolism developed during hospitalization: a nationwide retrospective observational study using claims data. *Cardiol. Ther.* **12**, 127–141 (2023). 36482141 10.1007/s40119-022-00290-6 PMC9734681

[27] Colak, E. et al. The RSNA pulmonary embolism CT dataset. *Radiol. Artif. Intell.* **3**, e200254 (2021). 33937862 10.1148/ryai.2021200254 PMC8043364

[28] Yang, X. et al. A Two-stage convolutional neural network for pulmonary embolism detection from CTPA images. *IEEE Access* **7**, 84849–84857 (2019).

[29] Shi, L. et al. Automatic Diagnosis of Pulmonary Embolism Using an Attention-guided Framework: A Large-scale Study. In *Proc. Third Conference on Medical Imaging with Deep Learning* 743–754 (PMLR, 2020).

[30] Huang, S. -C. et al. PENet-a scalable deep-learning model for automated diagnosis of pulmonary embolism using volumetric CT imaging. *NPJ Digit. Med.* **3**, 61 (2020). 32352039 10.1038/s41746-020-0266-y PMC7181770

[31] Rajan, D., Beymer, D., Abedin, S. & Dehghan, E. Pi-PE: A Pipeline for Pulmonary Embolism Detection using Sparsely Annotated 3D CT Images. In *Proc. of the Machine Learning for Health NeurIPS Workshop* 220–232 (PMLR, 2020).

[32] Suman, S. et al. Attention Based CNN-LSTM Network for Pulmonary Embolism Prediction on Chest Computed Tomography Pulmonary Angiograms. In *Medical Image Computing and Computer Assisted Intervention – MICCAI 2021: 24th International Conference, Strasbourg, France, September 27 – October 1, 2021, Proceedings, Part VII* 356–366 (Springer-Verlag, Berlin, Heidelberg, 2021). 10.1007/978-3-030-87234-2_34.

[33] Islam, N. U., Zhou, Z., Gehlot, S., Gotway, M. B. & Liang, J. Seeking an optimal approach for computer-aided diagnosis of pulmonary embolism. *Med. Image Anal.* **91**, 102988 (2024). 37924750 10.1016/j.media.2023.102988 PMC11039560

[34] Rathbun, S. W., Raskob, G. E. & Whitsett, T. L. Sensitivity and specificity of helical computed tomography in the diagnosis of pulmonary embolism: a systematic review. *Ann. Intern. Med.* **132**, 227–232 (2000). 10651604 10.7326/0003-4819-132-3-200002010-00009

[35] Stein, P. D. et al. Multidetector computed tomography for acute pulmonary embolism. *N. Engl. J. Med.* **354**, 2317–2327 (2006). 16738268 10.1056/NEJMoa052367

[36] Ghanima, W. et al. Multidetector computed tomography (MDCT) in the diagnosis of pulmonary embolism: interobserver agreement among radiologists with varied levels of experience. *Acta Radiol* **48**, 165–170 (2007). 17354136 10.1080/02841850601100859

[37] Nguyen, E. T. et al. Canadian Society of Thoracic Radiology/Canadian Association of Radiologists Best Practice Guidance for investigation of acute pulmonary embolism, Part 2: technical issues and interpretation Pitfalls. *Can. Assoc. Radiol. J.* **73**, 214–227 (2022). 33781102 10.1177/08465371211000739

[38] Sjöblom, T., Sladoje, N., Kahraman, A. T., Toumpanakis, D. & Fröding, T. Computed tomography pulmonary angiography (CTPA) data. *AIDA*10.23698/AIDA/CTPA (2019).

[39] Masoudi, M. et al. A new dataset of computed-tomography angiography images for computer-aided detection of pulmonary embolism. *Sci. Data* **5**, 180180 (2018). 30179235 10.1038/sdata.2018.180 PMC6122162

[40] Chen, Y. et al. SCUNet++: Swin-UNet and CNN Bottleneck Hybrid Architecture with Multi-Fusion Dense Skip Connection for Pulmonary Embolism CT Image Segmentation. In *2024 IEEE/CVF Winter Conference on Applications of Computer Vision (WACV)* 7744–7752 (IEEE, Waikoloa, HI, USA, 2024). 10.1109/WACV57701.2024.00758.

[41] Wasserthal, J. et al. TotalSegmentator: robust segmentation of 104 anatomic structures in CT images. *Radiol. Artif. Intell.* **5**, e230024 (2023). 37795137 10.1148/ryai.230024 PMC10546353

[42] Hahn, L. D., Hall, K., Alebdi, T., Kligerman, S. J. & Hsiao, A. Automated deep learning analysis for quality improvement of CT pulmonary angiography. *Radiol. Artif. Intell.* **4**, e210162 (2022). 35391776 10.1148/ryai.210162 PMC8980873

[43] Buslaev, A. et al. Albumentations: fast and flexible image augmentations. *Information* **11**, 125 (2020).

[44] Dai, Z., Liu, H., Le, Q. V. & Tan, M. CoAtNet: Marrying Convolution and Attention for All Data Sizes. In *Advances in Neural Information Processing Systems* (eds Ranzato, M., Beygelzimer, A., Dauphin, Y., Liang, P. S. & Vaughan, J. W.) Vol. 34, 3965–3977 (Curran Associates, Inc., 2021).

[45] Wightman, R. et al. rwightman/pytorch-image-models: v0.8.10dev0 Release. *Zenodo*10.5281/ZENODO.4414861 (2023).

[46] Sun, X. & Xu, W. Fast implementation of DeLong’s algorithm for comparing the areas under correlated receiver operating characteristic curves. *IEEE Signal Process. Lett.* **21**, 1389–1393 (2014).

