# Supplementary material for: High performance with fewer labels using semi-weakly supervised learning for pulmonary embolism diagnosis
Source: NPJ Digit Med. 2025 May 7;8:254. doi: 10.1038/s41746-025-01594-2 (PMC12059038; doi:10.1038/s41746-025-01594-2)
Supplement: Supplementary file 1 — Supplementary Materials [file 41746_2025_1594_MOESM1_ESM.pdf]

Supplementary Materials

Model Selection Criteria

Building on our team's previous work [1], which compared various feature extractor architectures including convolutional neural networks (CNNs) and vision transformers, we adopted CoAtNet as our primary model due to its high performance, pre-trained ImageNet weights, and computational efficiency. In our study, CoAtNet served as the main architecture, and we further validated our training strategy by testing it with ViT and CNNs, demonstrating its effectiveness across different model types. Results for these alternative feature extractors are provided in Supplementary Figure 3 and Supplementary Table 7.

[1] Phillips, P. *Automated Pulmonary Embolism Identification*. Master's thesis, University of Toronto (2023).

Supplementary Figure 1 ROC Curves Showing the Impact of Slice-Level Label Proportions on PE Detection Performance.

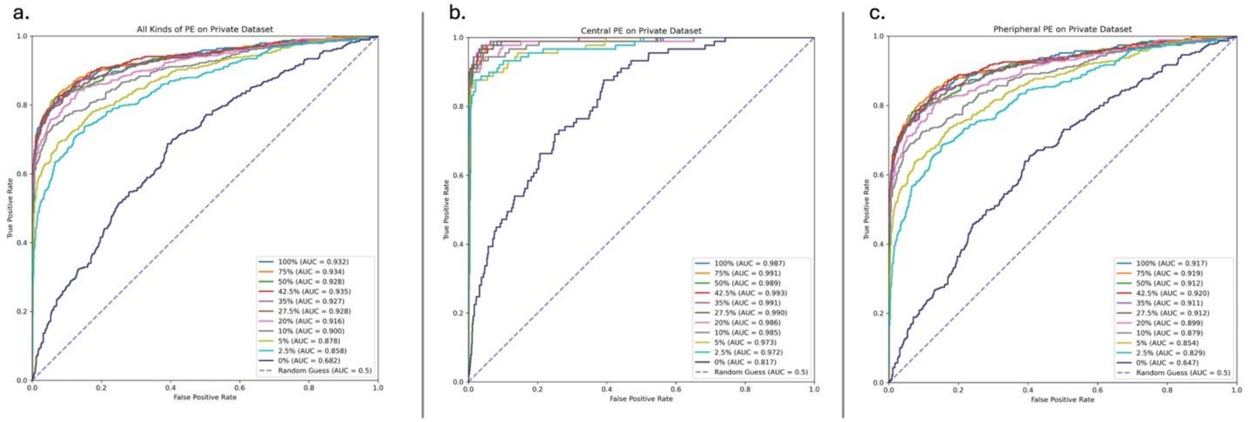

Receiver Operating Characteristic (ROC) curves for models trained with varying proportions of slice-level labels on the private test dataset. (a) Performance for all PE cases, (b) performance for central PE, and (c) performance for peripheral PE. Each curve represents a model trained with a different percentage of slice-level labels, illustrating how increasing label granularity affects diagnostic accuracy.

Supplementary Figure 2. Model Architectures for PE Diagnosis: Slice-Level, Exam-Level, and Hybrid Approaches

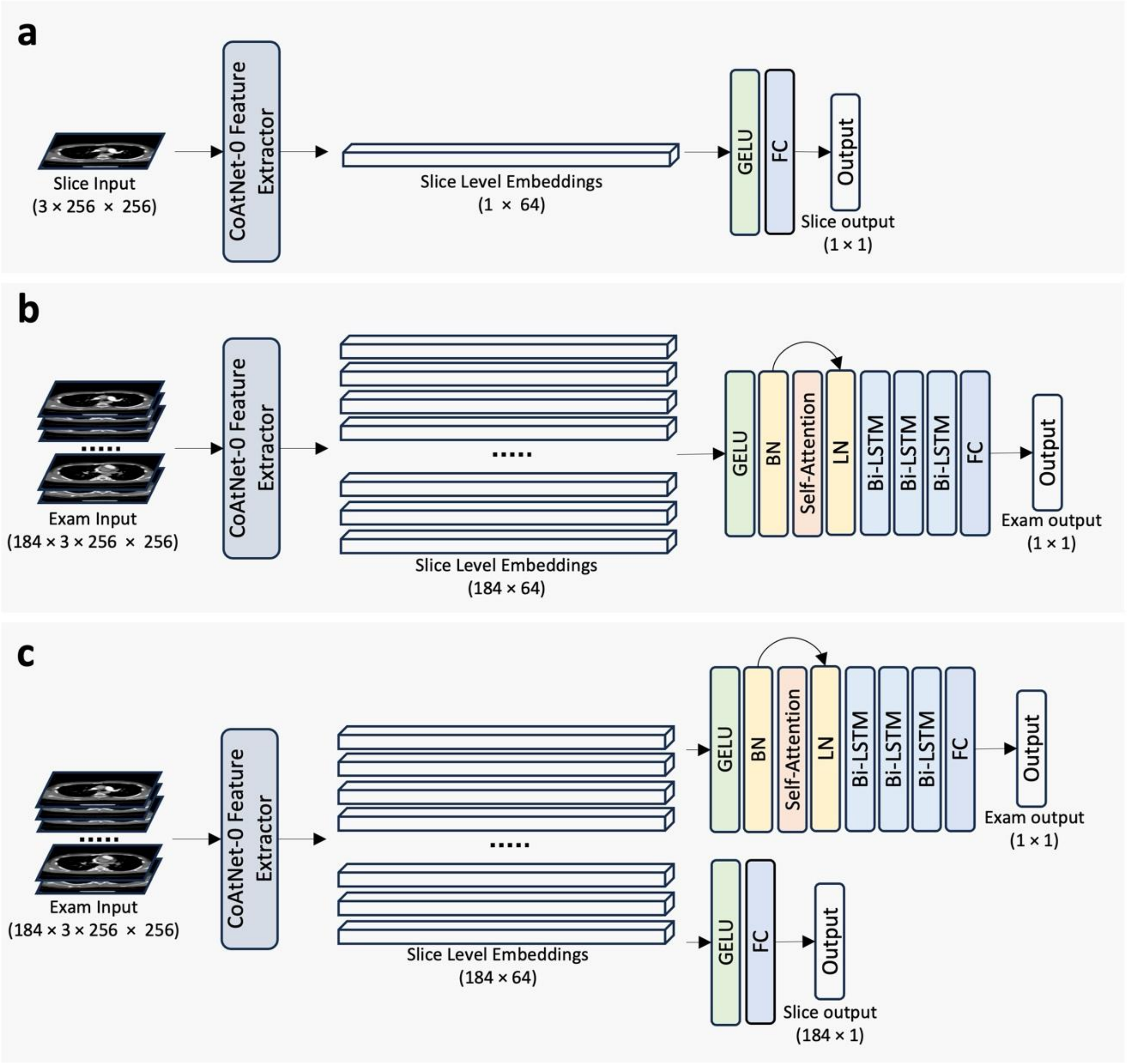

Model Architectures for PE Diagnosis: (a) Slice-Level Model: Trained on slice input with CoAtNet-0 extracting slice-level embeddings, followed by GELU and a fully connected (FC) layer for slice-level output. (b) Exam-Level Model: Trained on exam input with CoAtNet-0 extracting slice-level embeddings, processed by GELU, Batch Normalization (BN), Self-Attention, Layer Normalization (LN), and bidirectional LSTM (Bi-LSTM) layers, concluding with an FC layer for exam-level output. (c) Hybrid Model: Combines slice-level and exam-level training, starting with slice input processed by CoAtNet-0, followed by parallel paths for slice-level and exam-level embeddings, using layers

similar to the exam-level model for final outputs at both levels. Common practice in PE diagnosis involves using the slice-level model (a) and then aggregating with LSTM for exam-level prediction as in (b). Our hybrid model (c) improves training efficiency and ensures local slice-level information is retained. We found that when sufficient slice-level information is available (n > 30), the model can perform well by weighting the slice output loss at 0.95 and the exam output loss at 0.05

Supplementary Figure 3. Exam-Level AUC Trends Across Slice-Level Label Proportions for EfficientNet and Vision Transformer

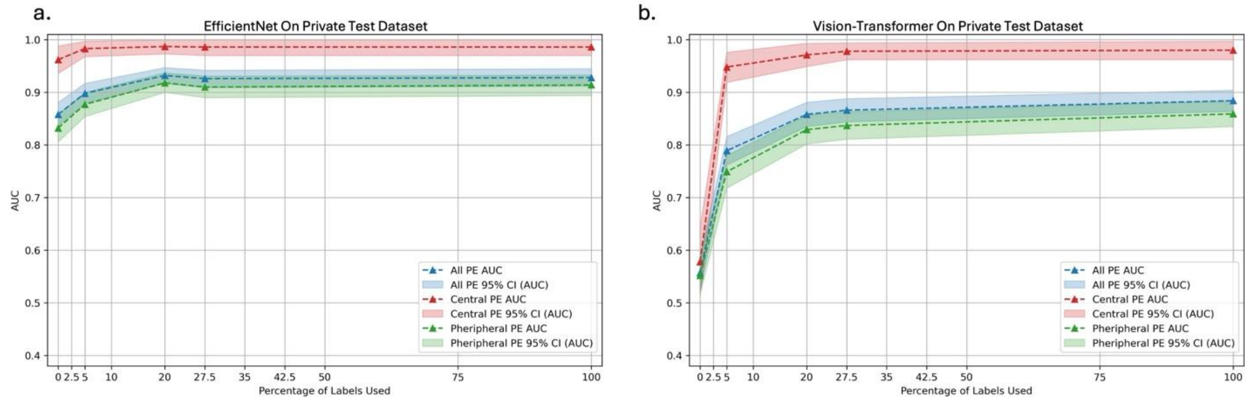

Exam-level AUC performance trends on the private test dataset for two architectures (a) EfficientNet and (b) Vision-Transformer across varying proportions of slice-level labels. Each line indicates the AUC for All PE, Central PE, and Peripheral PE detection, illustrating how increasing slice-level label availability affects model performance. Error bands represent the 95% confidence intervals.

Slice Labels (%)	RSPECT Public	RSPECT Private	External
100	0.932 (0.910, 0.955), --	0.932 (0.915, 0.948), --	1.000 (1.000, 1.000), --
75	0.937 (0.915, 0.958), 2.20e-01	0.934 (0.917, 0.950), 4.40e-01	0.998 (0.993, 1.000), 4.11e-01
50	0.931 (0.908, 0.953), 7.09e-01	0.928 (0.911, 0.945), 2.31e-01	0.999 (0.996, 1.000), 4.80e-01
42.5	0.928 (0.904, 0.951), 3.01e-01	0.935 (0.919, 0.951), 1.62e-01	0.998 (0.993, 1.000), 4.12e-01
35	0.925 (0.901, 0.950), 9.44e-02	0.927 (0.910, 0.944), 1.16e-01	0.996 (0.989, 1.000), 3.09e-01
27.5	0.925 (0.899, 0.950), 1.54e-01	0.928 (0.910, 0.945), 1.87e-01	0.999 (0.996, 1.000), 4.80e-01
20	0.917 (0.891, 0.943), 1.09e-02 *	0.916 (0.898, 0.934), 2.07e-05 *	0.996 (0.989, 1.000), 3.09e-01
10	0.909 (0.882, 0.936), 2.19e-03 *	0.900 (0.881, 0.920), 1.72e-08 *	0.993 (0.982, 1.000), 2.44e-01
5	0.875 (0.843, 0.908), 1.09e-06 *	0.878 (0.857, 0.899), 1.15e-11 *	0.977 (0.948, 1.000), 1.11e-01
2.5	0.863 (0.829, 0.897), 8.39e-09 *	0.858 (0.836, 0.881), 1.90e-15 *	0.980 (0.953, 1.000), 1.24e-01
0	0.720 (0.677, 0.764), 9.51e-22 *	0.682 (0.652, 0.711), 2.25e-56 *	0.656 (0.522, 0.790), 5.12e-07 *

Supplementary Table 1. Tabular data for model performance in terms of AUC across different datasets as a function of slice-level label availability. This table presents the AUC (95% CI) for average predictions of 5 fold cross validation models trained with varying percentages of slice-level labels (*0, 2.5, 5, 10, 20, 27.5, 35, 42.5, 50, 75%, 100%*) across RSPECT public, RSPECT private, and external datasets. The values shown are AUC (95% CI) for the corresponding percentage of slice-level labels used, along with p-values from the DeLong tests across the training replicates against the model using 100% of labels. Significant p-values ($p < 0.05$) are marked with an asterisk (*). '--' means not available. The 95% CI is calculated using DeLong Test across the average predictions of 5 CV experiments.

Slice Labels (%)	TP	FN	TN	FP	AUC	Acc	SEN	SEC	PPV	NPV	F1
0	151	41	252	198	0.720 (0.677, 0.764)	0.628 (0.590, 0.664)	0.786 (0.723, 0.839)	0.560 (0.514, 0.605)	0.433 (0.382, 0.485)	0.860 (0.816, 0.895)	0.558 (0.508, 0.608)
2.5	134	58	402	48	0.863 (0.829, 0.897)	0.835 (0.804, 0.862)	0.698 (0.630, 0.758)	0.893 (0.861, 0.919)	0.736 (0.668, 0.795)	0.874 (0.840, 0.901)	0.717 (0.663, 0.770)
5	136	56	423	27	0.875 (0.843, 0.908)	0.871 (0.843, 0.894)	0.708 (0.640, 0.768)	0.940 (0.914, 0.958)	0.834 (0.770, 0.884)	0.883 (0.851, 0.909)	0.766 (0.714, 0.818)
10	157	35	393	57	0.909 (0.882, 0.936)	0.857 (0.827, 0.882)	0.818 (0.757, 0.866)	0.873 (0.839, 0.901)	0.734 (0.671, 0.788)	0.918 (0.888, 0.941)	0.773 (0.725, 0.822)
20	145	47	432	18	0.917 (0.891, 0.943)	0.899 (0.873, 0.920)	0.755 (0.690, 0.811)	0.960 (0.938, 0.975)	0.890 (0.832, 0.929)	0.902 (0.872, 0.925)	0.817 (0.769, 0.864)
27.5	151	41	434	16	0.925 (0.899, 0.950)	0.911 (0.887, 0.931)	0.786 (0.723, 0.839)	0.964 (0.943, 0.978)	0.904 (0.850, 0.940)	0.914 (0.885, 0.936)	0.841 (0.796, 0.886)
35	151	41	426	24	0.925 (0.901, 0.950)	0.899 (0.873, 0.920)	0.786 (0.723, 0.839)	0.947 (0.922, 0.964)	0.863 (0.804, 0.906)	0.912 (0.883, 0.935)	0.823 (0.776, 0.869)
42.5	155	37	424	26	0.928 (0.904, 0.951)	0.902 (0.876, 0.923)	0.807 (0.746, 0.857)	0.942 (0.917, 0.960)	0.856 (0.798, 0.900)	0.920 (0.891, 0.941)	0.831 (0.786, 0.876)
50	151	41	421	29	0.931 (0.908, 0.953)	0.891 (0.864, 0.913)	0.786 (0.723, 0.839)	0.936 (0.909, 0.955)	0.839 (0.778, 0.885)	0.911 (0.882, 0.934)	0.812 (0.765, 0.859)
75	154	38	426	24	0.937 (0.915, 0.958)	0.903 (0.878, 0.924)	0.802 (0.740, 0.852)	0.947 (0.922, 0.964)	0.865 (0.807, 0.908)	0.918 (0.890, 0.940)	0.832 (0.787, 0.878)
100	151	41	431	19	0.932 (0.910, 0.955)	0.907 (0.882, 0.927)	0.786 (0.723, 0.839)	0.958 (0.935, 0.973)	0.888 (0.832, 0.927)	0.913 (0.884, 0.935)	0.834 (0.789, 0.880)

Supplemental Table 2. Performance in detecting PE on the RSPECT public test set. TP true positive, FN false negative, TN true negative, FP false positive, AUC area under the receiver operating curve, Acc accuracy, SEN sensitivity, SPEC specificity, PPV positive predictive value, and NPV negative predictive value.

Slice Labels (%)	TP	FN	TN	FP	AUC	Acc	SEN	SEC	PPV	NPV	F1
0	351	87	437	569	0.682 (0.652, 0.711)	0.546 (0.520, 0.571)	0.801 (0.761, 0.836)	0.434 (0.404, 0.465)	0.382 (0.351, 0.413)	0.834 (0.800, 0.863)	0.517 (0.485, 0.549)
2.5	313	125	871	135	0.858 (0.836, 0.881)	0.820 (0.799, 0.839)	0.715 (0.671, 0.755)	0.866 (0.843, 0.885)	0.699 (0.655, 0.739)	0.874 (0.852, 0.894)	0.707 (0.671, 0.742)
5	305	133	912	94	0.878 (0.857, 0.899)	0.843 (0.823, 0.861)	0.696 (0.652, 0.738)	0.907 (0.887, 0.923)	0.764 (0.720, 0.803)	0.873 (0.851, 0.892)	0.729 (0.693, 0.764)
10	344	94	860	146	0.900 (0.881, 0.920)	0.834 (0.814, 0.852)	0.785 (0.745, 0.821)	0.855 (0.832, 0.875)	0.702 (0.660, 0.741)	0.901 (0.881, 0.919)	0.741 (0.708, 0.775)
20	327	111	955	51	0.916 (0.898, 0.934)	0.888 (0.870, 0.903)	0.747 (0.704, 0.785)	0.949 (0.934, 0.961)	0.865 (0.827, 0.896)	0.896 (0.876, 0.913)	0.801 (0.769, 0.834)
27.5	344	94	954	52	0.928 (0.910, 0.945)	0.899 (0.882, 0.913)	0.785 (0.745, 0.821)	0.948 (0.933, 0.960)	0.869 (0.832, 0.898)	0.910 (0.891, 0.926)	0.825 (0.794, 0.856)
35	351	87	942	64	0.927 (0.910, 0.944)	0.895 (0.879, 0.910)	0.801 (0.761, 0.836)	0.936 (0.920, 0.950)	0.846 (0.808, 0.877)	0.915 (0.897, 0.931)	0.823 (0.792, 0.854)
42.5	364	74	915	91	0.935 (0.919, 0.951)	0.886 (0.868, 0.901)	0.831 (0.793, 0.863)	0.910 (0.890, 0.926)	0.800 (0.761, 0.834)	0.925 (0.907, 0.940)	0.815 (0.785, 0.846)
50	356	82	942	64	0.928 (0.911, 0.945)	0.899 (0.882, 0.913)	0.813 (0.774, 0.847)	0.936 (0.920, 0.950)	0.848 (0.810, 0.879)	0.920 (0.902, 0.935)	0.830 (0.800, 0.860)
75	367	71	921	85	0.934 (0.917, 0.950)	0.892 (0.875, 0.907)	0.838 (0.800, 0.869)	0.916 (0.897, 0.931)	0.812 (0.773, 0.845)	0.928 (0.911, 0.943)	0.825 (0.795, 0.855)
100	350	88	939	67	0.932 (0.915, 0.948)	0.893 (0.876, 0.908)	0.799 (0.759, 0.834)	0.933 (0.916, 0.947)	0.839 (0.801, 0.871)	0.914 (0.896, 0.930)	0.819 (0.788, 0.849)

Supplemental Table 3. Performance in detecting PE on the RSPECT private test set. TP true positive, FN false negative, TN true negative, FP false positive, AUC area under the receiver operating curve, Acc accuracy, SEN sensitivity, SPEC specificity, PPV positive predictive value, and NPV negative predictive value.

Slice Labels (%)	TP	FN	TN	FP	AUC	Acc	SEN	SEC	PPV	NPV	F1
0	37	1	1	26	0.656 (0.522, 0.790)	0.585 (0.463, 0.696)	0.974 (0.865, 0.995)	0.037 (0.007, 0.183)	0.587 (0.464, 0.700)	0.500 (0.095, 0.905)	0.733 (0.629, 0.836)
2.5	37	1	24	3	0.980 (0.953, 1.000)	0.938 (0.852, 0.976)	0.974 (0.865, 0.995)	0.889 (0.719, 0.961)	0.925 (0.801, 0.974)	0.960 (0.805, 0.993)	0.949 (0.879, 1.000)
5	36	2	24	3	0.977 (0.948, 1.000)	0.923 (0.832, 0.967)	0.947 (0.827, 0.985)	0.889 (0.719, 0.961)	0.923 (0.797, 0.973)	0.923 (0.759, 0.979)	0.935 (0.858, 1.000)
10	38	0	22	5	0.993 (0.982, 1.000)	0.923 (0.832, 0.967)	1.000 (0.908, 1.000)	0.815 (0.633, 0.918)	0.884 (0.755, 0.949)	1.000 (0.851, 1.000)	0.938 (0.865, 1.000)
20	38	0	25	2	0.996 (0.989, 1.000)	0.969 (0.895, 0.992)	1.000 (0.908, 1.000)	0.926 (0.766, 0.979)	0.950 (0.835, 0.986)	1.000 (0.867, 1.000)	0.974 (0.924, 1.000)
27.5	38	0	25	2	0.999 (0.996, 1.000)	0.969 (0.895, 0.992)	1.000 (0.908, 1.000)	0.926 (0.766, 0.979)	0.950 (0.835, 0.986)	1.000 (0.867, 1.000)	0.974 (0.924, 1.000)
35	38	0	25	2	0.996 (0.989, 1.000)	0.969 (0.895, 0.992)	1.000 (0.908, 1.000)	0.926 (0.766, 0.979)	0.950 (0.835, 0.986)	1.000 (0.867, 1.000)	0.974 (0.924, 1.000)
42.5	38	0	25	2	0.998 (0.993, 1.000)	0.969 (0.895, 0.992)	1.000 (0.908, 1.000)	0.926 (0.766, 0.979)	0.950 (0.835, 0.986)	1.000 (0.867, 1.000)	0.974 (0.924, 1.000)
50	38	0	25	2	0.999 (0.996, 1.000)	0.969 (0.895, 0.992)	1.000 (0.908, 1.000)	0.926 (0.766, 0.979)	0.950 (0.835, 0.986)	1.000 (0.867, 1.000)	0.974 (0.924, 1.000)
75	38	0	25	2	0.998 (0.993, 1.000)	0.969 (0.895, 0.992)	1.000 (0.908, 1.000)	0.926 (0.766, 0.979)	0.950 (0.835, 0.986)	1.000 (0.867, 1.000)	0.974 (0.924, 1.000)
100	38	0	25	2	1.000 (1.000, 1.000)	0.969 (0.895, 0.992)	1.000 (0.908, 1.000)	0.926 (0.766, 0.979)	0.950 (0.835, 0.986)	1.000 (0.867, 1.000)	0.974 (0.924, 1.000)

Supplemental Table 4. Performance in detecting PE on the external validation test set. TP true positive, FN false negative, TN true negative, FP false positive, AUC area under the receiver operating curve, Acc accuracy, SEN sensitivity, SPEC specificity, PPV positive predictive value, and NPV negative predictive value.

Slice Labels (%)	All PE AUC	Central PE AUC	Peripheral PE AUC
100	0.932 (0.915, 0.948), --	0.987 (0.974, 1.000), --	0.917 (0.898, 0.937), --
75	0.934 (0.917, 0.950), 4.40e-01	0.991 (0.980, 1.000), 1.35e-02 *	0.919 (0.899, 0.939), 6.46e-01
50	0.928 (0.911, 0.945), 2.31e-01	0.989 (0.975, 1.000), 2.59e-01	0.912 (0.892, 0.933), 1.61e-01
42.5	0.935 (0.919, 0.951), 1.62e-01	0.993 (0.986, 1.000), 3.96e-02 *	0.920 (0.900, 0.939), 3.66e-01
35	0.927 (0.910, 0.944), 1.16e-01	0.991 (0.978, 1.000), 2.65e-02 *	0.911 (0.890, 0.932), 5.88e-02
27.5	0.928 (0.910, 0.945), 1.87e-01	0.990 (0.977, 1.000), 1.49e-01	0.912 (0.891, 0.933), 1.19e-01
20	0.916 (0.898, 0.934), 2.07e-05 *	0.986 (0.971, 1.000), 6.57e-01	0.899 (0.877, 0.920), 1.54e-05 *
10	0.900 (0.881, 0.920), 1.72e-08 *	0.985 (0.971, 0.999), 5.60e-01	0.879 (0.855, 0.903), 1.17e-08 *
5	0.878 (0.857, 0.899), 1.15e-11 *	0.973 (0.957, 0.990), 4.31e-02 *	0.854 (0.829, 0.879), 2.55e-11 *
2.5	0.858 (0.836, 0.881), 1.90e-15 *	0.972 (0.953, 0.991), 5.05e-02	0.829 (0.802, 0.856), 2.85e-15 *
0	0.682 (0.652, 0.711), 2.25e-56 *	0.817 (0.776, 0.858), 1.75e-16 *	0.647 (0.614, 0.680), 6.10e-50 *

Supplementary Table 5. Exam-level AUC performance of models trained with varying proportions of slice-level labels for detecting all pulmonary emboli (All PE), central pulmonary emboli (Central PE), and peripheral pulmonary emboli (Peripheral PE). Values are presented as AUC (95% confidence interval) followed by the p-value from pairwise DeLong tests comparing each proportion to the fully supervised (100% slice labels) baseline. Asterisks (*) indicate statistically significant differences ($p < 0.05$).

Usage	Reason to exclude	Number of Exams
Train	ConversionError: MISSING_DICOM_FILES -- Missing slices (slice count mismatch between timepoint 0 and 1)	119
	ConversionValidationError: NON_CUBICAL_IMAGE/GANTRY_TILT -- Orthogonality check failed: non cubical image	35
	ConversionValidationError: SLICE_INCREMENT_INCONSISTENT -- Slice increment not consistent through all slices	10
	AttributeError: 'FileDataset' object has no attribute 'RepetitionTime'	3
	Mismatched Slice - Exam Label	153
	Misaligned Orientation	1
Public Test	impaired image quality issue	8
Private Test	impaired image quality issue	20
	ConversionError: MISSING_DICOM_FILES -- Missing slices (slice count mismatch between timepoint 0 and 1)	42
	ConversionValidationError: NON_CUBICAL_IMAGE/GANTRY_TILT -- Orthogonality check failed: non cubical image	9
	ConversionValidationError: SLICE_INCREMENT_INCONSISTENT -- Slice increment not consistent through all slices	2

Supplementary 6. Summary of exclusion criteria and the corresponding number of exams excluded from the training, public test, and private test datasets. Each row details a specific error or quality control issue (e.g., missing DICOM files, non-cubical image geometry, inconsistent slice increments, attribute errors, mismatched slice-exam labels, misalignment, or impaired image quality) that led to the removal of certain examinations. More detailed table can be found in https://github.com/zxjasonhu/pe_granular_analysis/blob/main/excluded_studies.csv

Architecture	Slice Labels (%)	All PE AUC	Central PE AUC	Peripheral PE AUC
EfficientNet	**100**	**0.928 (0.912, 0.945), --**	**0.986 (0.970, 1.000), --**	**0.914 (0.894, 0.933), --**
	27.5	0.926 (0.909, 0.942), 3.23e-01	0.986 (0.970, 1.000), 8.24e-01	0.910 (0.890, 0.931), 3.28e-01
	20	0.932 (0.917, 0.947), 3.36e-01	0.987 (0.973, 1.000), 8.21e-01	0.918 (0.900, 0.937), 3.44e-01
	5	0.898 (0.879, 0.917), 1.49e-06 *	0.983 (0.968, 0.997), 4.92e-01	0.877 (0.854, 0.900), 1.40e-06 *
	0	0.858 (0.836, 0.881), 5.05e-14 *	0.962 (0.936, 0.988), 3.93e-03 *	0.832 (0.806, 0.858), 3.63e-13 *
Vision-Transformer	**100**	**0.884 (0.864, 0.904), --**	**0.980 (0.962, 0.997), --**	**0.859 (0.835, 0.884), --**
	27.5	0.866 (0.844, 0.888), 5.50e-05 *	0.978 (0.962, 0.994), 7.57e-01	0.837 (0.811, 0.863), 3.82e-05 *
	20	0.858 (0.835, 0.881), 2.07e-08 *	0.971 (0.949, 0.993), 1.55e-01	0.829 (0.802, 0.857), 3.84e-08 *
	5	0.789 (0.762, 0.816), 1.92e-21 *	0.948 (0.919, 0.976), 7.97e-04 *	0.749 (0.718, 0.780), 2.39e-20 *
	0	0.557 (0.525, 0.589), 8.32e-80 *	0.578 (0.512, 0.643), 2.05e-32 *	0.552 (0.517, 0.586), 4.69e-60 *

Supplementary Table 7. Exam-level AUC performance of two different architectures (EfficientNet and Vision-Transformer) trained with varying proportions of slice-level labels for detecting all pulmonary emboli (All PE), central pulmonary emboli (Central PE), and peripheral pulmonary emboli (Peripheral PE). Values are presented as AUC (95% confidence interval) followed by p-values from pairwise DeLong tests comparing each proportion to the fully supervised (100% slice labels) baseline. Asterisks (*) indicate statistically significant differences ($p < 0.05$).

%of slice-level labels	Mean Slice Annotations (Positive and Negative) per exam
0	0.0±0.0
2	3.9±0.9
5	9.7±2.2
10	19.4±4.5
20	38.8±9.0
27.5	53.4±12.3
35	67.9±15.7
42.5	82.5±19.0
50	97.0±22.4
75	145.5±33.6
100	194.0±44.8

Supplementary Table 8. Average number of slice labels available per exam (mean ± standard deviation) at different percentages of slice-level label utilization. The average lung length segmented by TotalSegmentator is 193.96 ± 44.81 slices.
